# Plant responses and rhizosphere soil characteristics of sea-buckthorn from different sex combinations in an abandoned lead-zinc mine

**DOI:** 10.3389/fpls.2025.1601834

**Published:** 2025-07-31

**Authors:** Tingjiang Gan, Zhenxiong Zeng, Wenhao Pei, Qi Jia, Yunxiao He, Juan Chen

**Affiliations:** ^1^ School of Urban and Rural Planning and Construction, Mianyang Teachers’ College, Mianyang, China; ^2^ College of Architecture, Changsha University of Science & Technology, Changsha, China

**Keywords:** dioecious plant, sexual difference, abandoned mine, soil properties, nutrient content, eco-physiological responses

## Abstract

**Background:**

Nowadays, the restoration of abandoned mines has gained more attention due to its significance in vegetation recovery and ecological security Although some studies have indicated the sexual dimorphism of dioecious plants in response to the environmental adaptability, sea-buckthorn (*Hippophae rhamnoides* L.), a diecious species widely used in afforestation and soil conservation, has not been studied the effects of sexual interactions on degraded ecosystem restoration;

**Methods:**

*In situ* experiment, the physiological responses and rhizosphere soil changes of sea-buckthorn seedlings from different sex combinations were investigated in an abandoned Lead-Zinc mine;

**Results:**

The two sexes from intra- and inter-sexual combinations showed differences in chlorophyll content, antioxidant activities, carbohydrates, proline, nutrient elements and zinc content in plants, and total organic carbon, nutrient elements and enzyme activities in rhizosphere soils. The males from the inter-sex combination had stronger antioxidant capacity and more osmoregulatory substances in plants and soil nitrogen contents as well as significantly higher activities of protease and urease with the increases of 139%, and 56%, respectively. In the intra-sex combination, compared with male plants, female plants showed higher contents of total N, Zn, soluble sugar and starch in the roots increased by 30.3%, 75%, 41.5% and 93.7% respectively, as well as higher soil available phosphate and potassium.

**Conclusions:**

Sexual combinations significantly affected plant responses and soil properties of *H. rhamnoides* in the abandoned mine, male plants showed better adaptability than female plants in inter-sex combination, while females showed better rhizosphere responses than male plants in same-sex combination, which suggests that sexual interactions of dioecious species should be considered in the restoration of degraded ecosystem.

## 1 Introduction

Although mineral exploitation has brought great economic benefits, the abandoned mining land had led to serious environmental pollution and ecological destruction. The vegetation destruction, soil pollution and erosion caused by mining pose a major threat to biological health and ecosystem stability (Xu et al., 2023; Setu and Strezov, 2025). Therefore, all countries around the world attach great importance to the restoration of abandoned mining areas (Ortega-Larrocea et al., 2010; Hendrychová et al., 2020). Common restoration techniques include physical, chemical and biological restorations and their combination. It is well known that physical and chemical remediation methods have many limitations, including irreversible changes in soil properties and soil microflora as well as secondary pollution, etc (Sikdar et al., 2020). Bioremediation, especially phytoremediation technology, has the advantages of simple operation technology, low cost, small disturbance to the environment, and can be deployed in a large area (Liang et al., 2011). In addition to improving soil fertility and soil environment, increasing species diversity, it also rebuild ecological landscape (Ortega-Larrocea et al., 2010). Therefore, vegetation restoration is considered to be the most effective way to improve the ecological environment of mining areas (Yuan et al., 2023). Woody plants, in particular, can effectively reduce environmental risks caused by heavy metal migration due to developed roots, large biomass, strong nutrient retention and *in-situ* fixation of heavy metals (Castiglione et al., 2009; Kim et al., 2018).

Dioecious plants are an important component of terrestrial ecosystems and play an active role in maintaining species diversity and ecosystem stability (Renner, 2014). It was reported that dioecious plants show sex differences under environmental stresses such as heavy metals, drought and salt stress etc. Most studies have found that male plants have better response mechanism and stronger adaptability to poor habitats (Dolkar et al., 2017; Liu et al., 2020), but there are also studies that show the opposite (Sánchez-Vilas and Retuerto, 2009; Gao et al., 2010), which suggests that sexual dimorphic responses are related to species and stress factors. Sea-buckthorn (*Hippophae rhamnoides* L.), a diecious species belonging to the genus of *Hippophae*, family of Elaeagnaceae, is an important tree species for ecological protection (Gao et al., 2010). It can stabilize soil and reduce erosion because of its advantages of strong adaptability, easy reproduction, better resistances to drought, cold and barren environment (Lin et al., 2023; Zhang et al., 2022). Sea-buckthorn has been widely used in afforestation, soil conservation and nitrogen fixation in barren habitats (He et al., 2013; Zhao et al., 2025).

Plant interactions including facilitation and competition are of great significance to vegetation reconstruction and ecological restoration under stress environment (Song et al., 2022). Stress gradient hypothesis (SGH) indicates that the competitiveness between plants turns into promotion with the enhancement of environmental stress (Bertness and Callaway, 1994). Previous researches suggest that SGH is universal on a global scale, especially in the higher environmental pressure (Gómez-Aparicio, 2009; Nemer et al., 2022), but some studies are opposed to the SGH hypothesis (Noumi, 2020; Tanner et al., 2023). It can be seen that plant interaction profoundly affects the adaptability of individual plants to the environment. As is well known, a large number of abandoned mining areas suffer from nutrient element deficiency and heavy metal pollution in the soil, which leads to dual environmental stresses on plant growth. The selection of species with large differences in functional traits is an efficient and low-cost method to restore degraded ecosystems, so as to minimize competition and maximize promotion (Navarro-Cano et al., 2019).

Previous study pointed out that the quantities of soil microorganisms and nutrient contents varied when the sea buckthorn combined with different plant species in the open-pit mine dump (Ma et al., 2007).

Recent years, some studies mainly focused on the sex ratio, morphology, growth and physiological differences of sea-buckthorn in various environment. The sea-buckthorn had sexual differences in leaf and palisade tissue thickness, and the main vein vascular bundle of male plant was more developed (Li et al., 2017). Male plants of sea-buckthorn had a better self-protection mechanism than female plants under cold and freezing conditions (Dolkar et al., 2017). However, female plants of *H. rhamnoides* showed stronger adaptability and physiological adjustment to drought stress, with more proline (Pro) accumulation, stable protective enzyme system and lower lipid membrane peroxidation (Gao et al., 2010).

Moreover, male-female interaction and manganese stress interactively affected chlorophyll, antioxidant enzyme activities and osmoregulatory substances in leaves, and soil microorganism of sea-buckthorn (Fang et al., 2022; Lin et al., 2023). Therefore, it can be seen that the sexual difference and interactions between two sexes may affect its adaptation to various environment, which may influence the ecological restoration effect of dioecious plants on degraded habitats. However, there are relatively few researches to explore the effect of sex combinations on the process of vegetation restoration in abandoned mining land.

The response of plant rhizosphere under environmental stress and the synergistic feedback mechanism with aboveground parts have been paid more attention. Soil enzyme activities and nutrient elements are very sensitive to various vegetation restoration model and land use change. Most of the studies are conducted in the greenhouse about the responses of plants to heavy metal stress (Chen et al., 2011; Liu et al., 2020), but the *in-situ* restoration experiments on abandoned land are relatively few (Yan et al., 2020; Song et al., 2022). An *in-situ* remediation experiment of long-term contaminated soil showed that polychlorinated biphenyls and heavy metals in soil were significantly reduced after poplar planting, and the abundance and diversity of rhizosphere microorganisms generally increased (Ancona et al., 2021). In the restoration experiments of Cd contaminated land, the bacterial community richness in rhizosphere soil of *Populus deltoides* was significantly higher than that in bare soil, and endogenous fungi enriched metabolites related to plant signaling compounds and nucleotides (Zhang et al., 2022). Therefore, the plant responses and soil properties should be connected to exactly evaluate the plant adaptation and restoration effect in degraded ecosystems. In this study, three different sex combinations of female and female (FF), male and male (MM) and female and male (FM) of *H. rhamnoides* were set on the abandoned Lead-Zinc mines to explore the plant physiological parameters and rhizosphere soil response characteristics. The study is aimed to answer the questions: (1) Do sex combinations affect the physiological response process, soil enzymes and nutrient status of male and female plants of *H. rhamnoides* in the *in-situ* restoration of abandoned mine experiment? (2) Whether male and female plants in the intra-sex and inter-sex combination show different competitive pressure and adaptation abilities in the Lead-Zinc abandoned mine? Under which sex combination do female plants or male plants exhibit better adaptability? (3) What correlation do exist between the plant responses and the rhizosphere soil characteristics when evaluating the adaptability of *H. rhamnoides* to abandoned mine under sex combinations? The results will prompt research about dioecious plant adaptability and interactions under stress environment, and provide scientific basis for the application of sea-buckthorn in ecological restoration of abandoned mining land.

## 2 Materials and methods

### 2.1 Plant materials and experimental design

The experiment was arranged at the Lead-Zinc mine located at Loufanggou, Xiangyan Town, Pingwu county of Sichuan province. The area has a humid subtropical monsoon climate, with annual average temperature of 16 °C, annual average rainfall of 840 mm. In the experiment, the sea-buckthorn seedlings with a height of approximately 40cm, good growth and uniform morphology, were planted in the abandoned mine according to different sex combinations respectively. This experiment was set up with a completely random grouping. Three combinations were conducted as follows, male plant-only (MM), female plant-only(FF), or mixed female and male plant (FM) in the experiment, and each combination was planted in four plots, and about 40 seedlings were planted in each plot (the width and length were both 2 meters). After planting, watering and weeding was performed regularly to ensure the normal growth of seedlings. After six months of growth, four male and female seedlings were randomly selected from each treatment plot and carefully removed from the field soil. The whole plant was sampled and divided into three parts: root, stem (including main branch and lateral branch) and leaf. The plant roots were gently shaken to remove most of the non-root zone soil, while retaining the soil aggregates (0.5~5mm) that is closely attached to the root surface as rhizosphere soil. The collected rhizosphere soil sample was placed in a dry ice box and brought back to the laboratory for further processing. Part of the collected leaves were stored in a -80°C for the determination of the content of chlorophyll and proline (Pro) as well as protective enzyme activities. The remaining leaves, roots and stems were cleaned and dried to constant weight at 70°C, crushed through a sieve with an aperture of 80 mesh size, and used for the determination of soluble sugar (SS), starch and nutrient elements in plants. Rhizosphere soil samples were dried by natural draft to determine soil enzyme activities and nutrient elements.

### 2.2 Determination of leaf biochemical parameters

The 0.3g cut fresh leaves were grinded with a small amount of calcium carbonate, quartz sand and 5ml of 80% acetone to extract the pigments. Then the extracting solution was filtered and the volume set to 25ml using 80% acetone, and the absorbance was measured at 645 and 663 nm with a spectrophotometer. The chlorophyll a (Chl a) and chlorophyll b (Chl b) contents were calculated using the method described by Lichtenthaler (1987). The determination of proline content in leaves was referred to the method described by Bates et al. (1973). The content of Malondialdehyde (MDA) was determined by thiobarbituric acid method. The 0.1g fresh leaves were homogenized in 10 ml of 10% trichloroacetic acid and centrifuged at 4°C for 10 min at 4000r/min. The supernatant was collected and mixed with 2 ml of 0.6% thiobarbituric acid and heated in a water bath at 100°C for 15 min. After cooling, the mixture was centrifuged at 4000r/min for 15 min. Then the absorbances of the supernatant at 450 nm, 600nm and 532 nm was determined (Fang et al., 2024).The calculation formula is as follows: MDA (mol·g^-1^ FW) = $\frac{[6.45(A532-A600)-0.56A450]\text{V}t}{Vs*FW}$ . In formula, Vt: total volume of extract (ml); Vs: the volume of the extract that used for determination (ml); FW: fresh weight of sample (g).The 0.3g fresh leaves were grinded in liquid nitrogen and extracted with a 50 mM potassium phosphate buffer (pH 7.8) containing 0.1 mM EDTA, 1% (w/v) polyvinylpyridone (PVP), 0.1 mM phenylmethylsulfonyl fluoride (PMSF) and 0.2% (v/v) Triton X-100 for the measurement of superoxide dismutase (SOD) and peroxidase (POD). The SOD activity was determined by measuring its ability to inhibit nitro blue tetrazolium (NBT) photoreduction, and absorbance was measured at 560nm by a spectrophotometer. The amount of enzyme required to inhibit 50% of the NBT photoreduction reaction was taken as a unit of enzyme activity (U·g^-1^FW·min^-1^) (Chen et al., 2011). Prepare a reaction mixture containing 50 mM potassium phosphate buffer (pH 6.0), 10 mM hydrogen peroxide, 40 mM guaiacol, and 0.1ml of enzyme extract for measuring POD activity at 470 nm using a spectrophotometer. Measure the absorbance at 470 nm every 30 seconds within 3 minutes. The activity of POD is calculated using the extinction coefficient of the oxidation guaiacol product, and the unit is U·g^-1^FW·min^-1^ (Chen et al., 2011; Hao et al., 2020).

### 2.3 Determination of plant nutrient elements and carbohydrate content

Take 0.5 g of the plant sample and place it in a digestion tube. Add an appropriate amount of concentrated sulfuric acid and hydrogen peroxide to digest sample, ensuring complete dissolution. Transfer the digested solution to a volumetric flask, dilute it to the volume to 50 mL. Then, the 5ml solution was drawn for the content of total nitrogen in plants determined by ultraviolet spectrophotometry after potassium persulfate oxidation method (Lv et al., 2004). The total phosphorus (P) content in plants was determined by the molybdenum-antimony colorimetric method. Take 5 mL of the digestion solution, adding 5 mL of molybdenum- antimony chromogenic reagent into a volumetric flask, then dilute to 50 mL to measure the absorbance of the solution at 700 nm. Potassium (K) and zinc (Zn) in plants were determined by the flame spectrophotometry. The emission light intensity of the digested sample solution was measured at 766.5 nm of K wavelength and 213.9 nm of Zn wavelength respectively. Calculate the K and Zn contents through the standard curve. The translocation factor (TF) was the ratio of Zn concentration in leaf and stem to root Zn. The contents of plant soluble sugar and starch were determined by anthrone method, and absorbances were recorded at 630 nm according to the methods of Hedge et al. (1962) and Fang et al. (2024). Take 0.1 g of dried samples of plant roots, stems and leaves, dissolve them in 5 ml of 80% alcohol, and perform water bath at 80°C for 40 minutes. After cooling and centrifugation, extract the residue multiple times and combine the supernatants. The supernatant was added the anthrone reagent to determine the soluble sugar content. Then, add 9.2 mmol·L^−1^ and 4.6 mmol·L^−1^ perchloric acid respectively to the residues for extraction. Combine the supernatants and dilute to 50 ml, add the anthrone reagent to determine the starch content.

### 2.4 Soil enzyme activity and nutrient element

The soil organic carbon (TOC) was measured using external heated potassium dichromate oxidation method (Nelson and Sommers, 2018). Soil nitrogen, phosphorus and potassium were prepared by H_2_O_2_-H_2_SO_4_ dehydrating method for testing, and the content of total nitrogen (TN) in soil was determined by indophenol blue colorimetry. The contents of total phosphorus (TP) and available phosphorus (AP) were determined by molybdenum-antimony resistance colorimetry. Soil nitrate nitrogen (NO_3_^–^ N) and ammonium nitrogen (NH_4_^+^ -N) were extracted by 2 mol·L^−1^ potassium chloride and measured using colorimetry assay (Bao, 2000). Soil potassium (K) was determined by flame spectrophotometry. The dried soil sample was extracted by water (the ratio of soil to water is 1:2.5), and was determined by an Acidimeter (PHS-3C; LEICI, Shanghai). With reference to the method described by Guan (1986), soil enzyme activity was determined. The 3, 5-dinitrosalicylic acid colorimetry was used to determine sucrase (SC), and its activity was expressed as the mass (mg) of glucose released by 1 g of soil after 1 day. Urease (UE) was determined by phenol-sodium hypochlorite colorimetric method, and its activity was expressed as NH_3_-N mass (mg) released by 1 g of soil after 1 day. Neutral phosphatase (NP) was determined by phenylene disodium phosphate colorimetric method, and its activity was expressed as phenol release mass (mg) in 1 g soil after 1 day. Protease (PT) was determined by ninhydrin colorimetry, and its activity was expressed by the mass of glycine (mg) released in 1 g of soil after 1 day. Catalase (CAT) enzyme activity was determined by potassium permanganate titration. The soil sample was add 3% (v/v) hydrogen peroxide and placed at 4°C for 1 hour. After filtrating, the supernatant was added 0.2 mol·L^−1^ sulfuric acid, and was titrated using 0.1 mol·L^−1^ potassium permanganate standard solution. The CAT activity was expressed as the mass (mg) of 1 g of soil decomposed hydrogen peroxide in 1 minute.

### 2.5 Data analysis

In this study, IBM SPSS Statistics 22 was used to make descriptive statistics on the data, and one-way ANOVA was used to process the data to test the significant differences of each observation index under different sex combination modes (*P*<0.05). The *post-hoc* test of one-way ANOVA was conducted by Duncan method. The principal component analysis (PCA) was conducted to determine the main physiological response parameters of plants and soil biochemical parameters in principal components, and analyze the response differences of two sexes under sexual combinations in an abandoned mine.

## 3 Results

### 3.1 Differences in leaf physiological response parameters

**Figure 1:**
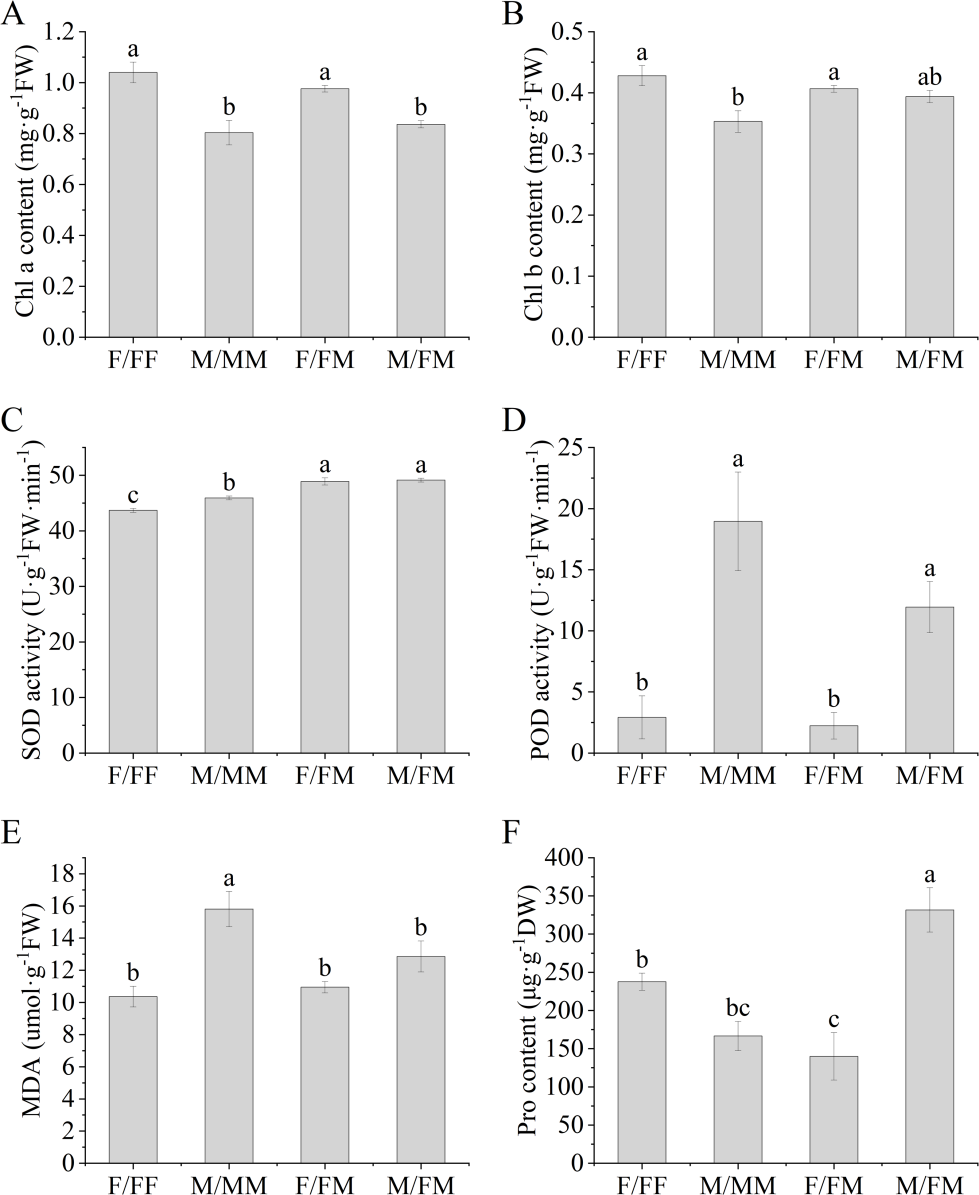
Leaf physiological response parameters of female and male *H. rhamnoides* seedlings under sexual combinations in the Lead-Zinc mine. **(A)**, Chla content; **(B)**, Chlb content; **(C)**, SOD activity; **(D)**, POD activity; **(E)**, MDA; **(F)**, Pro content. Different lowercase letters mean significant differences under different sex combinations, according to Duncan’s test (*P*< 0.05). Values are means ± SE (n = 4). F/FF, soil from the female–female intrasex combination treatment; M/MM, soil from the male–male intrasex combination treatment; F/FM and M/FM, soil from the female–male intersex combination treatment.

As shown in **Figure 1**, in the intra-sex combination, the contents of Chl a and Chl b in F/FF were significantly higher than those in M/MM, with increases of 30% and 22.9%, respectively. In the intersex combination, the content of Chla in F/FM was significantly 16.7% higher than that in M/FM (**Figures 1A, B**). Compared with the intra-sex combination, the SOD activity of two sexes was significantly higher under the inter-sex combination (**Figure 1C**). Among all sex combination, the POD activity of male plants was significantly higher than that of female plants. Under intersexual competition, the Pro content and POD activity in male plants were 137% and 435% higher, respectively, than those in female plants (**Figures 1D, F**). For female plants, F/FF showed higher Pro content and lower SOD activity than that of F/FM. For male plants, M/FM showed higher SOD activity and Pro content, while lower MDA content than M/MM (**Figures 1C, E, F**).

### 3.2 Differences in plant carbohydrates and nutrient content

**Figure 2:**
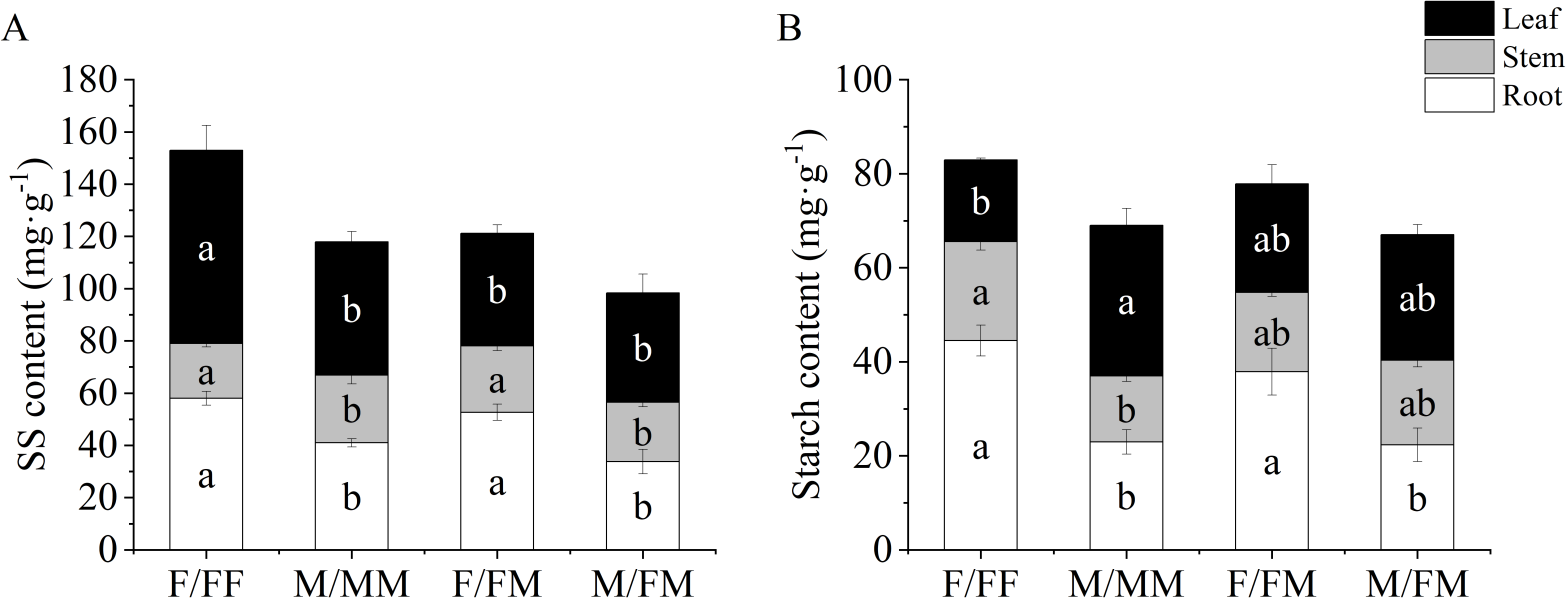
Carbohydrates in roots, stems and leaves of female and male *H*. *rhamnoides* seedlings under sex combinations in Lead-Zinc mine. **(A)**, SS content; **(B)**, Starch content. Different lowercase letters mean significant differences under different sex combinations, according to Duncan’s test (*P* < 0.05). Values are means ± SE (n = 4).

In the intra-sex combination, F/FF showed higher SS content in roots, stems and leaves and starch content in roots and stems, while lower starch content in leaves than that in M/MM (**Figures 2A, B**). The contents of SS in roots and stems and starch content in roots of F/FM were significantly higher than those of M/FM by 55.8%, 12.1%, and 69.6%, respectively, in the inter-sex combination. Compared with female plants in intrasex combination, the SS content in the leaves of female plants in the intersex combination was decreased by 14.9% (**Figure 2A**).

**Figure 3:**
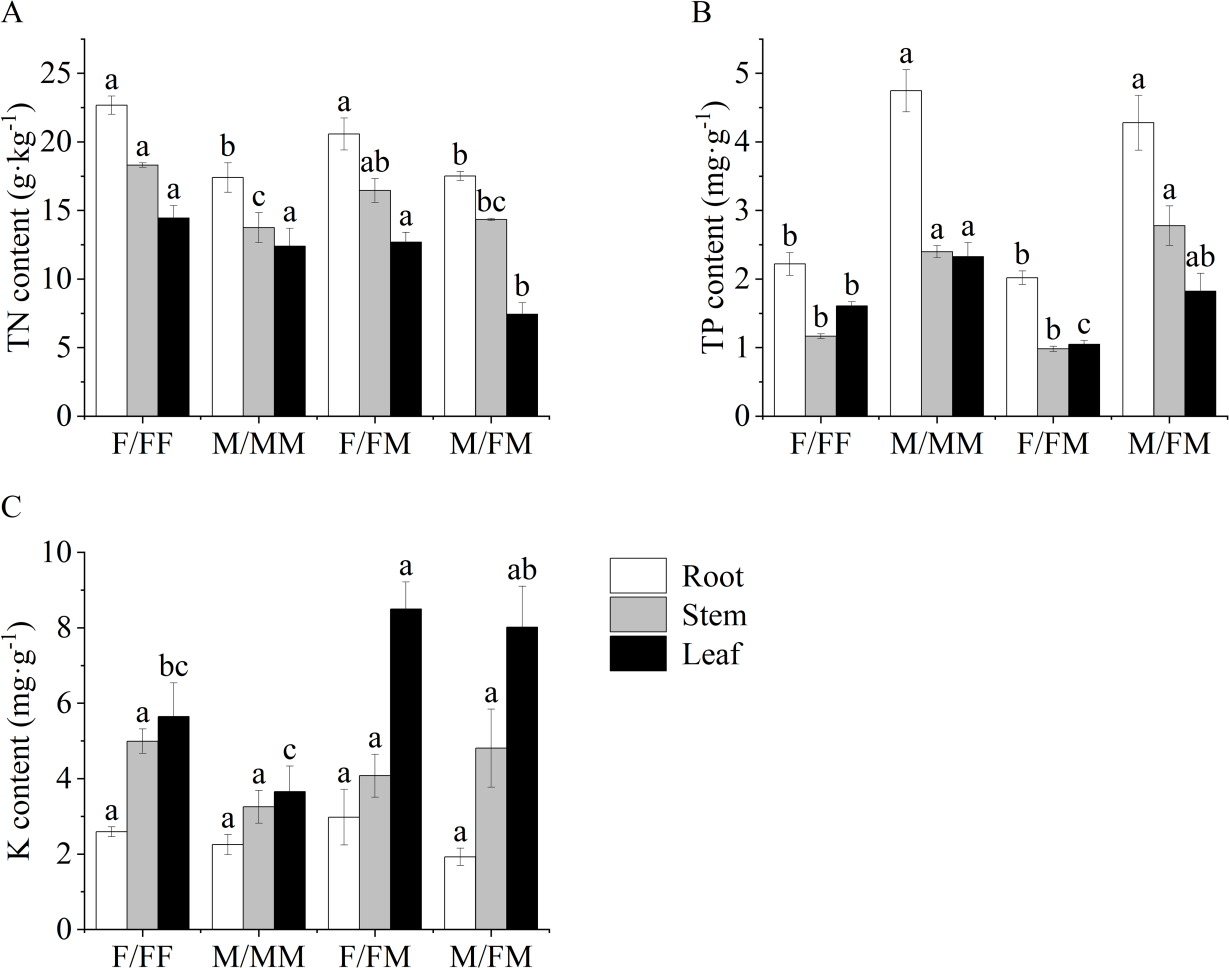
Nutrient contents of roots, stems and leaves of female and male *H*. *rhamnoides* seedlings under sex combinations in Lead-Zinc mine. **(A)**, TN content, Total nitrogen content; **(B)**, TP content, total phosphorus content; **(C)**, K content, potassium content. Different lowercase letters mean significant differences under sex combinations according to Duncan’s test (p < 0.05). Values are means ± SE (n = 4).

**Figure 4:**
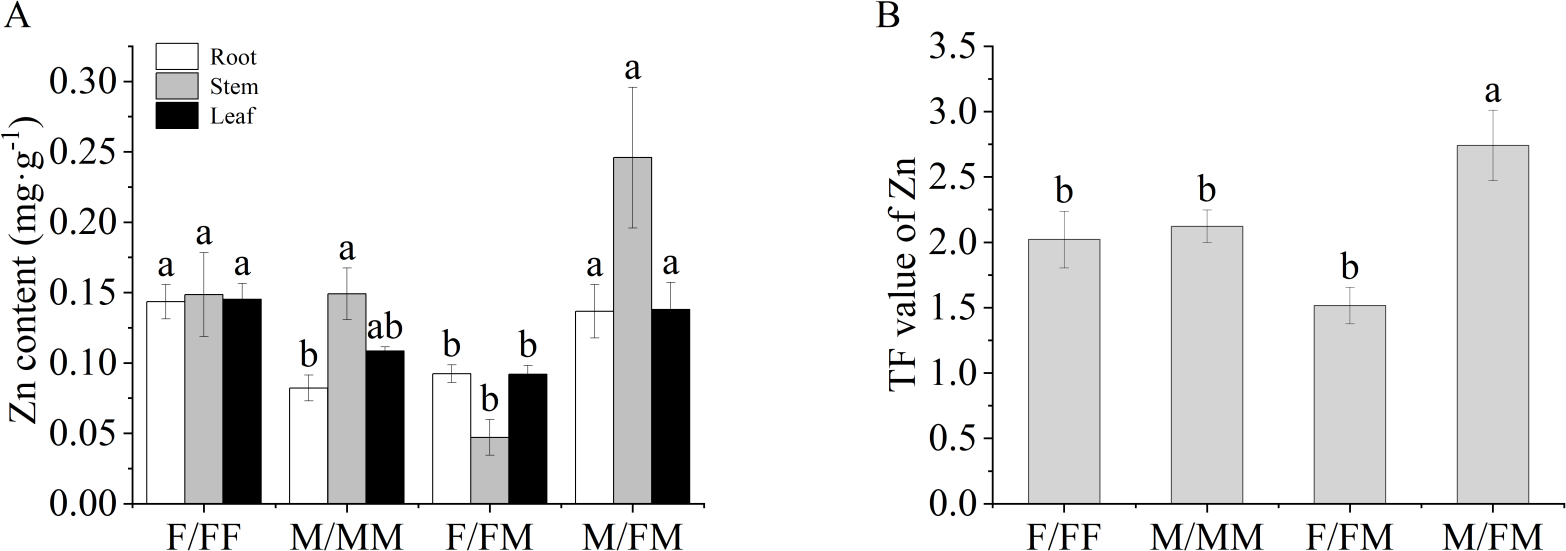
Zn contents of roots, stems and leaves and TF value of female and male *H*. *rhamnoides* seedlings under sex combinations in Lead-Zinc mine. **(A)**, Zn content, Zinc content, **(B)**, TF value, translocation factor. Different lowercase letters mean significant differences under sex combinations according to Duncan’s test (p < 0.05). Values are means ± SE (n = 4).

As shown in **Figure 3**, compared with M/MM, the TN in the roots and stems of F/FF increased by 30.3% and 33.2%, respectively. The Zn in the roots of F/FF increased by 75%, while the P content in the roots decreased by 114% (**Figures 3A, B**, **4A**). In the inter-sex combination, TN content in roots and leaves of F/FM was significantly higher than that in M/FM by 17.5% and 70.7%, respectively (**Figure 3A**), while TP and Zn content in all organs of M/FM were significantly higher than those in F/FM (**Figures 3B**, **4A**). The content of K in leaves of both two sexes in intersex combination was significantly higher than that in intra-sex combination (**Figure 3C**). Compared with F/FF, F/FM had lower leaf TP and Zn content in all organs (**Figures 3B**, **4A**). The TF value of Zn of M/FM was the highest among all sex combinations (**Figure 4B**).

### 3.3 Difference in soil enzyme activity and chemical properties

**Figure 5:**
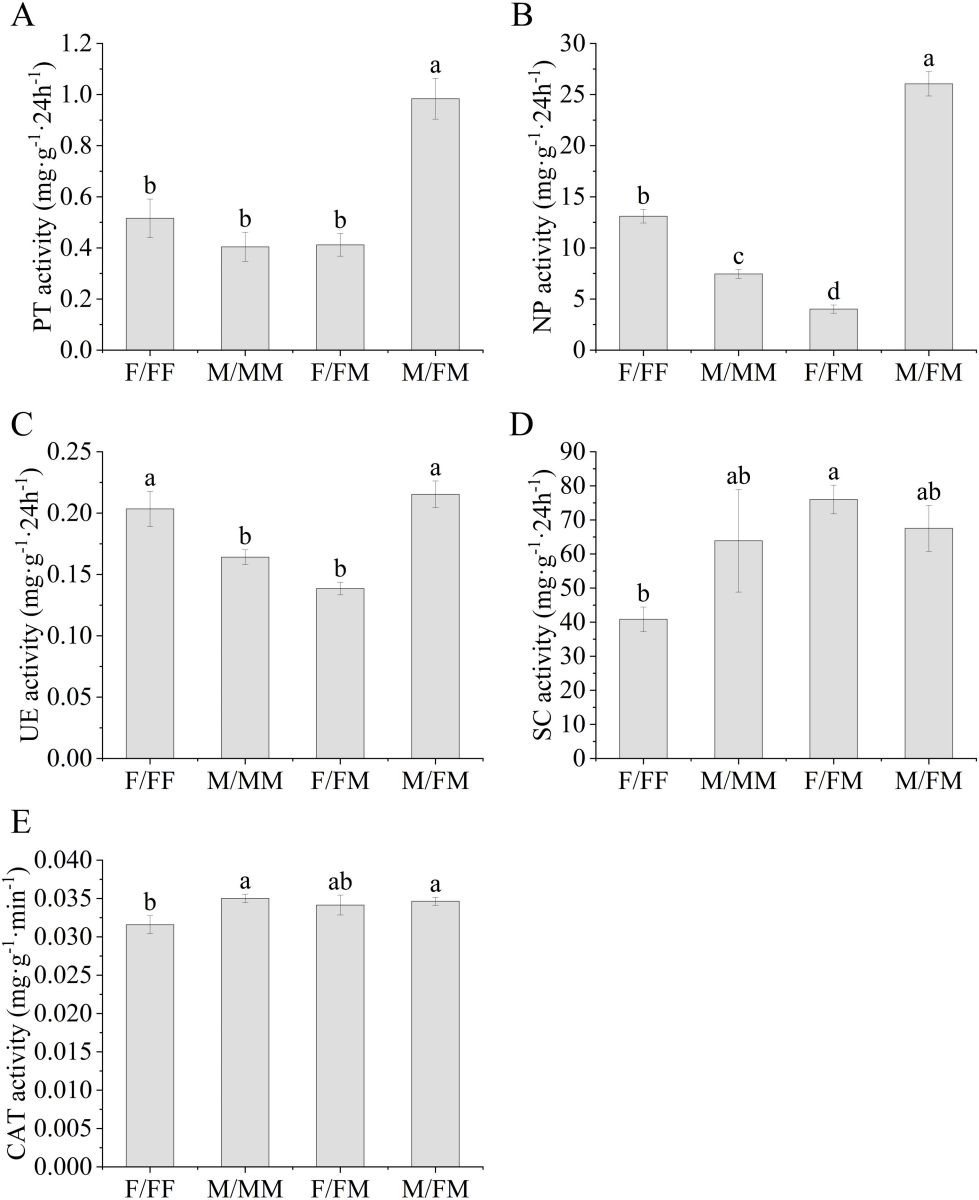
Enzyme activities in rhizosphere soil of *H*. *rhamnoides* under different sex combinations in the Lead-Zinc mine. **(A)**, PT activityProtease activity; **(B)**, NP activityNeutral phosphatase activity; **(C)**, UE activityUrease activity; **(D)**, SC activitySucrase activity; **(E)**, CAT activityCatalase activity. Different lowercase letters mean significant differences under different sex combinations, according to Duncan’s test (p < 0.05). Values are means ± SE (n = 4).

As shown in **Figure 5**, the rhizosphere soil PT and NP activities of M/FM was the highest among all sex combinations (**Figures 5A, B**). In the intra-sex combination, UE activity of F/FF was significantly higher than that of M/MM, with increase of 25%, while CAT activity of M/MM was significantly higher than that of F/FF (**Figures 5C, E**). In the intersex combination, PT, NP and UE activities of M/FM were significantly higher than those of F/FM, with increase of 139%, 549% and 56%, respectively. The SC activity of F/FM was 86% higher than F/FF (**Figure 5D**). The activities of PT, NP and UE in the rhizosphere soil of M/FM were higher than those of M/MM, with increase of 145%, 250% and 31%, respectively, while NP and UE activities of F/FM were lower than F/FF, with decrease by 69% and 31% (**Figures 5A–C**).

As shown in **Table 1**, in the inter-sex combination, the TOC content of M/FM was 31% lower than that of F/FM, but the contents of TN, NH_4_^+^-N and NO_3_^–^N were 85%, 92%, and 110% higher than that of F/FM, respectively. In the intra-sex competition, compared with male plants, the available phosphorus and potassium contents in the rhizosphere soil of female plants increased by 29% and 64%, respectively, while the NO_3_^–^N content decreased by 44%. The F/FM had the highest AP content, while M/MM showed the lowest AP content among all sex combinations.

**Table 1:** Soil physicochemical properties under different sex combinations of *H. rhamnoides* in abandoned Lead-Zinc mining area.

Soil properties	F/FF	M/MM	F/FM	M/FM
pH	6.71 ± 0.02b	6.86 ± 0.01ab	6.73 ± 0.01b	6.75 ± 0b
TOC (g·kg^-1^)	22.88 ± 1.56a	27.41 ± 3.17a	22.87 ± 2.05a	15.85 ± 0.61b
TN (g·kg^-1^)	0.22 ± 0.04b	0.21 ± 0.05b	0.21 ± 0.03b	0.39 ± 0.07a
NH_4_^+^-N (mg·kg^-1^)	2.78 ± 0.24b	2.27 ± 0.12b	2.20 ± 0.16b	4.23 ± 0.31a
NO_3_^–^N (mg·kg^-1^)	0.29 ± 0.03c	0.52 ± 0.02a	0.19 ± 0.06c	0.40 ± 0.03b
TP (g·kg^-1^)	0.33 ± 0.03a	0.28 ± 0.02a	0.29 ± 0.02a	0.31 ± 0.02a
AP (mg·kg^-1^)	5.84 ± 0.21b	4.52 ± 0.35c	6.87 ± 0.19a	5.77 ± 0.25b
K (g·kg^-1^)	3.57 ± 0.25a	2.17 ± 0.37c	2.64 ± 0.14bc	3.13 ± 0.13ab

^1^ : TOC, Total organic carbon content; TN, Total nitrogen content; NH_4_^+^-N (mg/kg), Ammonium nitrogen content; NO_3_^–^N, Nitrate nitrogen content; TP, Total phosphorus content; AP, Available phosphorus content; K, Total potassium content. In the same row, different lowercase letters mean significant differences under different combinations in the abandoned Lead-Zinc mine (p < 0.05). Values are means ± SE (n = 4). F/FF, soil from the female-female intrasex combination; M/MM, soil from the male-male intrasex combination; F/FM and M/FM, soil from the female-male intersex combination respectively.

### 3.4 PCA

**Figure 6:**
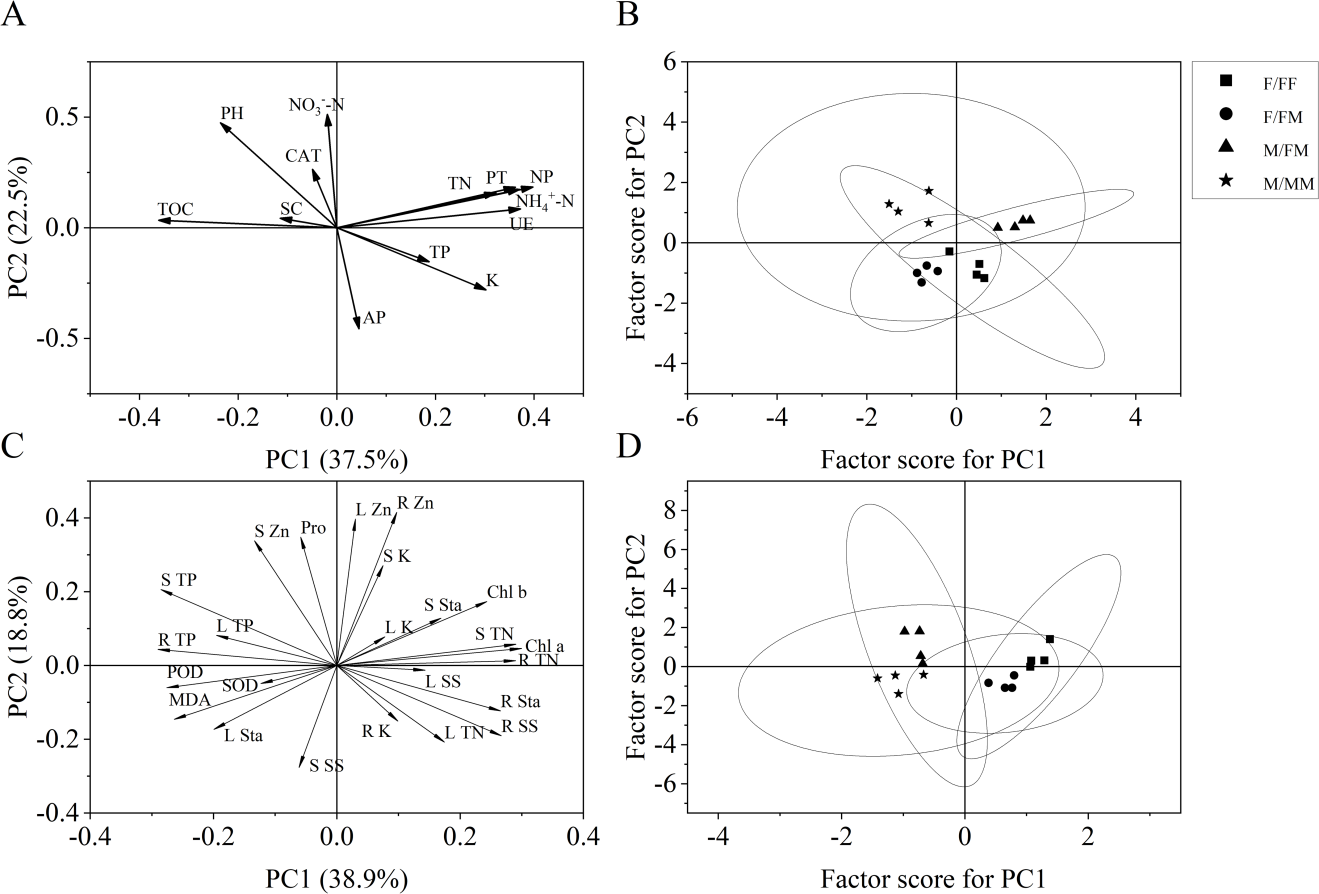
Results of the principal component analysis (PCA). **(A)** Correlations of factor loadings of soil properties of *H. rhamnoides* seedlings. **(B)** Soil factor scores for different sex combinations under the Lead-Zinc mine. **(C)** Correlations of factor loadings of plant physiological indicators of *H. rhamnoides* seedlings. **(D)** Plant factor scores for different sex combinations under the Lead-Zinc mine. The variable acronyms are as follow: TOC, total organic carbon; TN, total nitrogen content; TP, total phosphorus content; NH_4_^+^-N, ammonium nitrogen content; NO_3_^–^N, nitrate nitrogen content; AP, available phosphorus content; K, total potassium content; PT, protease activity; NP, neutral phosphatase activity; UE, Urease activity; SC, Sucrase activity; CAT, catalase activity; Chl a, Chlorophyll a content; Chl b, Chlorophyll b content; SOD, peroxide dismutase activity; POD, peroxidase activity; MDA, malondialdehyde; Pro, proline content; LTN, leaf total nitrogen content; STN, stem total nitrogen content; RTN, root total nitrogen content; LTP, leaf total phosphorus content; STP, stem total phosphorus content; RTP, root total phosphorus content; LK, leaf potassium content; SK, stem potassium content; RK, root potassium content; LZn, leaf zinc content; SZn, stem zinc content; RZn, root zinc content; LSS, leaf soluble sugar content; SSS, stem soluble sugar content; RSS, root soluble sugar content; LSta, leaf starch content; SSta, stem starch content; RSta, root starch content.

The two-component PCA model based on soil properties explained 60% of the variation under different sex combinations. The first component PC1 (37.5%) was positively correlated with NP, PT, UE, NH_4_^+^-N, TN, TP, K and AP, while negatively correlated with NO_3_^–^N, CAT, pH, SC and TOC. The PC1 was more affected by PT, NP, TN, NH_4_^+^-N, UE and K (**Figure 6A**). The results of factor analysis based on rhizosphere soil response parameters showed that the distance difference and separation degree between the F/FF, F/FM and M/MM were not significant and could be merged into one group, while the difference between M/FM and other sexual combinations was significant. Also, M/FM showed higher scores on PC1, while M/MM had higher scores on the PC2 axis (**Figure 6B**). The second component (22.5%) was mainly affected by pH, the contents of NO_3_^–^N and TOC. The two-component PCA model based on plant parameters explained 57.7% of the variation under different sex combinations, and the first component (38.9%) PC1 was greatly influenced by Chla, Chlb, STN, RTN, Rsta and RSS, while the second component (18.8%) is mainly affected by LZn, RZn, SZn, Pro and LK (**Figure 6C**). Factor analysis based on plant response parameters showed that F/FF had a higher score on the PC1 axis, while M/FM had higher score on the PC2 axis (**Figure 6D**). The differences and separation between sex combinations showed that the difference between F/FF and F/FM was small and could be merged into one group, while the M/MM and M/FM could be merged into one group.

## 4 Discussion

### 4.1 Effect of sex combinations on plant biochemical responses

Plants can resist the heavy metal stress by adjusting the antioxidant system and osmoregulatory substances. MDA can reflect the degree of damage of cell membranes. Antioxidant enzymes such as SOD and POD can eliminate reactive oxygen species and protect cells from oxidative damage (Chen et al., 2011). Our results showed significant responses of antioxidant enzymes, chlorophyll, carbohydrates and nutrient elements of sea-buckthorn in the abandoned Lead-Zinc mines. Previous studies found that male poplar in intersexual competition showed higher osmotic regulation ability, water use efficiency and antioxidant enzyme activity to salt stress (Li et al., 2016), and the sexual interaction model significantly affected the photosynthetic parameters, carbon and nitrogen balance, ultrastructure and enrichment capacity of *Populus cathayana* under heavy metal stress (Chen et al., 2016; 2017). It was reported that the SOD, POD activities and MDA contents of two sexes of sea-buckthorn showed significant sexual differences under the different sex combinations and manganese stress (Fang et al., 2022). Our results also found that SOD activity and K content in leaves of both two sexes in intersexual combination were significantly higher than that of two sexes in intrasexual combination. In the experiment, F/FM showed higher Chl a, SS in roots and stems and starch in roots, TN in roots and leaves, while lower Pro, TP and Zn content in all organs and POD activity compared with that of M/FM. The results indicated that female plants had stronger photosynthetic capacity, more carbohydrates and nitrogen accumulation, while male plants showed stronger antioxidant capacity and more accumulation of osmoregulatory, zinc and phosphorus in inter-sex combination in Lead-Zinc abandoned mine. Some studies indicated that DNA methylation-mediated metabolism of phenylpropane and starch prompted male poplar more tolerant to nitrogen stress, and male plants showed higher osmoregulation ability and resistance gene expression (Song et al., 2019; 2020). Therefore, it can be speculated that the sexual-specific responses of *H. rhamnoides* might be related to the changes in resistance gene expression, plant hormones and metabolism affected by environmental stresses and combination patterns. On the other hand, F/FF had higher contents of Chl a and Chl b, SS in roots, stems and leaves and starch in roots and stems, TN in roots and stems and Zn in roots than that in M/MM, indicating that female plants had stronger photosynthetic capacity and more osmoregulatory substances and zinc accumulation than male plants under the intra-sex combination in the Lead-Zinc mine. Moreover, F/FF showed higher Pro and SS content in the leaves, leaf TP and Zn content in all organs, while lower SOD activity than that of F/FM, indicating that female plants under intra-sex combination accumulated more osmoregulatory substances and nutrient elements. For male plants, M/FM had higher SOD activity and Pro content and lower MDA than that of M/MM, as well as the highest TF value among all sex combinations, indicating better adaptation in abandoned Lead-Zinc mine. It was reported that *Populus tremula* had a sexual trade-off between growth, salicylate and flavonoid-derived phenylpropyl production, with females preferentially accessing mineral nutrients, producing flavonoids and concentrated tannins (Randriamanana et al., 2014). So, our results also found the sexual trade-off between mineral nutrients and carbohydrates that caused by different sexual neighbors in the abandoned Lead-Zinc mine. Previous studies indicated that male poplar had higher heavy metal accumulation and better adaptation than female plants under nutrient deficiency and Cd stress (Chen et al., 2011; Liu et al., 2020). However, our results indicated that the sexual-specific differences was also related to the different neighbors within the sex combinations. The sex interaction may affect the response process of female plants and male plants to environmental stress. The differences in the photosynthesis, antioxidant enzymes, osmotic regulation and nutrient absorption of two sexes of sea-buckthorn may imply the sexual trade-off between growth and defense under sex combinations and the abandoned Lead-Zinc mine.

### 4.2 Effect of sex combinations on responses of rhizosphere soil

The rhizosphere is the interface between roots and the soil where nutrient absorption for plant growth. An abundant and diverse rhizosphere biome is involved in biogeochemical processes, driving soil C, N and P dynamics (Larsen et al., 2015). Soil enzymes are mainly derived from the activities of soil microorganisms, plant root exudates and decomposed animal and plant residues. Soil sucrase, protease and phosphatase are closely related to the carbon, protein decomposition, nitrogen and phosphorus cycles, while soil catalase can alleviate the toxicity of hydrogen peroxide in soil (Burke et al., 2011; Feng et al., 2025). In our study, rhizosphere soil properties of two sexes also showed sexual differences in the abandoned Lead-Zinc mine. The soil phosphatase and urease activities of female plants were higher than that of male plants in intra-sex combination, indicating that higher organophosphorus and urea decomposition ability, thus improving the availability of soil nutrients. It was found that different sex combinations under the manganese stress resulted in significant changes in the activities of sucrase, urease and phosphatase in the rhizosphere soil of male and female sea-buckthorn (Lin et al., 2023). Our study have observed the different responses of two sexes when interacting different sex neighboring plants. Compared with intra-sexual combination, M/FM had higher contents of soil TN, NH_4_^+^-N, AP, K, PT, NP and UE, while F/FM showed lower NP and UE activities, indicating that sex combination patterns significantly affected soil nutrient and enzymes. In addition, male plants had stronger soil enzymes activities and better decomposition ability of protein, urea and organophosphorus in inter-sexual combination under the abandoned Lead-Zinc mine. It was reported that the phenol metabolites in the roots of female plants were more varied between intersexual and intrasexual interactions, and the mixed rhizosphere of *Populus cathayana* showed higher diversities of microbial, bacterial and fungal communities (Xia et al., 2022). So, the relatively high nitrogen content in the rhizosphere soil of M/FM may be related to the abundant microbial communities that may promote nitrogen absorption and transformation. Previous study found that the functional flora of rhizosphere soil of two sexes of sea-buckthorn showed sexual differences, which further affected the heavy metal tolerance to Mn stress (Lin et al., 2023). Therefore, in our study, the differences in soil enzymes and nutrients of two sexes from sex combinations may imply the changes in rhizosphere microbe community.

### 4.3 Interaction between plant and rhizosphere soil under sex combinations

It is well known that the interaction between the plant and rhizosphere soil affects the response process and overall adaptability of plants to the environment (Larsen et al., 2015; Xia et al., 2022). In this study, in the inter-sex combination, female plants of *H. rhamnoides* showed higher carbohydrates in roots and soil total organic carbon, indicating that female plants allocate more photosynthates in roots and prefer to soil carbon-related metabolisms, whereas, male plants had stronger antioxidant capacity and more osmoregulatory substances in aboveground part, and higher soil nitrogen contents and activities of soil protease, neutral phosphatase and urease, indicating that male plants tend to improve soil nitrogen fixation and phosphorus absorption by way of decreasing the plant oxidant pressures and increasing the soil enzyme activities in response to the abandoned mine. Previous studies indicated that *H. rhamnoides* showed significant sex differences in rhizosphere soil microbe under manganese stress (Lin et al., 2023), and two sexes had the sexual differences in physiological responses when inoculating with AMF (Arbuscular Mycorrhizal Fungi) under the Pb and Zn stress (Fang et al., 2024). It was reported that plants regulated the structure and function of rhizosphere soil microorganisms through litter decomposition and root exudates, thus affecting soil nutrient transformation and ecosystem carbon and nitrogen cycling (Xia et al., 2022; Guo et al., 2024). Therefore, in our study, the responses of plant and rhizosphere soil of *H. rhamnoides* may be related to the changes of soil microbe and root exudates under sex combinations in abandoned mine environment, but it needs further study. Previous studies indicated that the sex interaction patterns of dioecious plants affect the ability of roots to capture resources (Sánchez-Vilas et al., 2011; Rogers and Eppley, 2012). In brief, the interaction between plant and rhizosphere soil as well as the trade-off of resources allocation caused by sex combinations may affect the adaptation and potential remediation of two sexes of *H. rhamnoides* and the ecosystem nutrient cycling in the abandoned mine. The PCA of soil parameters explained 60% of variation, while the PCA of plant parameters explained 57.7% of the variation in different experiment treatments. The PCA based on soil parameters distinguished M/FM from other sex combinations, while the PCA based on plant physiological parameters differentiated two sexes in sex combinations, which may suggest different competition pressure on two sexes in intra-and inter-sexual combinations. It was reported that *Antennaria dioica* had higher intensity of intra-sex competition in female plants, which resulted in asymmetric niche isolation between the sexes (Varga and Kytöviita, 2012). The sex composition and interaction pattern of *Populus cathayana* plantation regulated the enzyme activity related to soil nitrogen cycle, and the intersex interaction reduced the ecological niche overlap and resource competition (Guo et al., 2024). In the experiment, different sex combinations may imply different competitive pressure and interactive effect on two sexes, which affect the adaptation to the adverse habitat. The male plants from inter-sex combination had higher adaptation and restoration potential than female plants, while female plants in the intra-sex combination accumulated total nitrogen, Zn, carbohydrates in roots as well as soil AP and K content. Therefore, the appropriate sex combination can improve the efficiency of ecological restoration of sea-buckthorn in abandoned Lead-Zinc mine. In the future, many studies should be attention on the divergent effects of sex combination varying with different plant varieties or heavy metal-contaminated environments.

## 5 Conclusions

The study showed that sex combinations affected the physiological response, soil enzymes and nutrient status of male and female plants of *H. rhamnoides* in the *in-situ* restoration experiment on an abandoned Lead-Zinc mine. In the abandoned lead-zinc mine site, female and male plants from both same-sex and opposite-sex combinations exhibited differences in terms of competitive pressure and adaptability. The male plants from the intersex combination had stronger activities of antioxidant enzymes and more osmoregulatory substances in plants, as well as higher soil nitrogen and the activities of protease, neutral phosphatase and urease, indicating better bioavailability of decomposing protein and transforming organophosphorus. In intra-sex combination, female plants showed stronger photosynthetic capacity, osmoregulatory substances and zinc accumulation in roots than male plants. This indicates that in same-sex and opposite-sex combinations affect their responses and adaptation abilities to the abandoned mine. Our results provided the new insight of the adaptation and remediation potential of dioecious plants in the abandoned mine. These findings underscore the importance of considering sexual dimorphism in the selection of restoration species for degraded mine. Therefore, sex interactions of dioecious species should be considered in the restoration of degraded ecosystem in the future.

## Data Availability

The raw data supporting the conclusions of this article will be made available by the authors, without undue reservation.
